# Mechanisms of Upconversion Luminescence of Er^3+^-Doped NaYF_4_ via 980 and 1530 nm Excitation

**DOI:** 10.3390/nano11102767

**Published:** 2021-10-19

**Authors:** Yu Liu, Ziwen Zhou, Shaojian Zhang, Enming Zhao, Jing Ren, Lu Liu, Jianzhong Zhang

**Affiliations:** 1 Key Laboratory of In-Fiber Integrated Optics of Ministry of Education, College of Physics and Optoelectronic Engineering, Harbin Engineering University, Harbin 150001, China; thuliuyu2022@163.com (Y.L.); zw543202021@163.com (Z.Z.); yanyi0131@hrbeu.edu.cn (S.Z.); ren.jing@hrbeu.edu.cn (J.R.); liulu@hrbeu.edu.cn (L.L.); 2 School of Engineering, Dali University, Dali 671003, China; zhaoem163@163.com

**Keywords:** Er^3+^, upconversion, NaYF_4_, 980 and 1530 nm excitation, mechanisms

## Abstract

To date, the mechanisms of Er^3+^ upconversion luminescence via 980 and 1530 nm excitation have been extensively investigated; however, based on discussions, they either suffer from the lack of convincing evidence or require elaborated and time-consuming numerical simulations. In this work, the steady-state and time-resolved upconversion luminescence data of Er^3+^-doped NaYF_4_ were measured; we therefore investigated the upconversion mechanisms of Er^3+^ on the basis of the spectroscopic observations and the simplified rate equation modeling. This work provides a relatively simple strategy to reveal the UCL mechanisms of Er^3+^ upon excitation with various wavelengths, which may also be used in other lanthanide ion-doped systems.

## 1. Introduction

Rare earth ion (Re^3+^)-doped materials emitting upconversion luminescence (UCL) have attracted increasing attention owing to their wide application potentials in super-resolution nanoscopy [1], visible and ultraviolet lasers [2], 3-D volumetric display [3], information security [4], photovoltaic devices [5], cancer therapy [6], anti-counterfeiting [7], and biological fluorescence labeling [8]. Appropriate hosts and activators are of vital importance for achieving desired UCL performances. Besides its good stability validating its use in various conditions, hexagonal NaYF_4_ (*β*-NaYF_4_), with the maximum phonon energy below 400 cm^−1^ [9] diminishing the phonon-assisted relaxation of excited electrons and thereby increasing the light emission intensity, has become the most popular UCL host.

**Figure 1:**
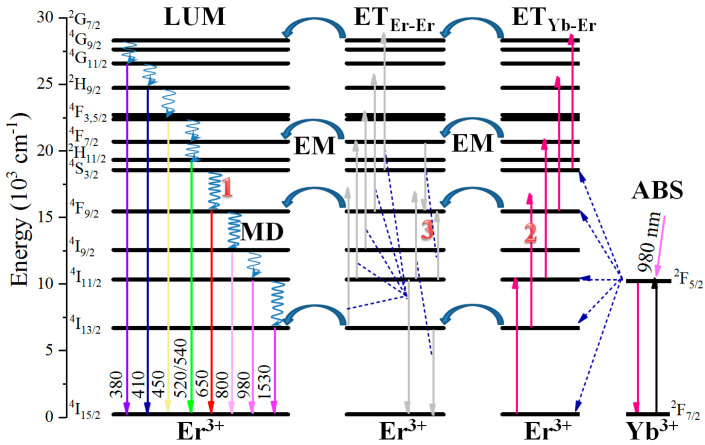
Diagram of Yb^3+^ and Er^3+^ energy levels with the main possible pathways involved in the Er^3+^ luminescence processes. ABS, absorption; ET_Yb-Er_, ET from Yb^3+^ to Er^3+^; EM, energy migration between neighboring Er^3+^; ET_Er-Er_, ET within the levels of the same Er^3+^ ion; MD, multiphonon-assisted decay; LUM, luminescence.

From another side, Er^3+^ is the most attractive activator for UCL, mainly due to the high luminescent efficiency and the abundant light colors including the RGB components [10]. As shown in Figure 1, traditional Er^3+^ doped UCL materials, usually with the help from Yb^3+^ as sensitizer, are mostly irradiated at ~980 nm [11]. Yb^3+^-sensitized Er^3+^ UCL exhibits higher efficiency, owing to Yb^3+^ holding a large absorption cross-section at 980 nm (~9×10^−21^ cm^2^ for Yb^3+^ and ~2 × 10^−21^ cm^2^ for Er^3+^) [12,13] and can efficiently transfer the energy absorbed to Er^3+^, enabling Er^3+^ luminescence ranging from ultraviolet to visible and to NIR. The mechanisms of Er^3+^ luminescence have been extensively investigated in Er^3+^/Yb^3+^ co-doped materials, comprising mainly the absorption of Yb^3+^, energy transfer (ET) processes (from Yb^3+^ to Er^3+^, among different Er^3+^ ions, or within the levels of the same Er^3+^ ion), multiphonon-assisted decays, and finally the spontaneous radiative transitions (Figure 1). However, the UCL mechanisms of Er^3+^ upon 980 nm excitation seem to be sensitive to many factors, especially for the red emission. To date, the origins of red UCL of Er^3+^ upon 980 nm excitation are generally attributed to the following three processes as labeled in Figure 1: 1. the multiphonon-assisted decay from the upper state [14], 2. the upward transition from ^4^I_13/2_ state [15], 3. the energy transfer (ET) within the levels of the same Er^3+^ [16]. How to distinguish the dominant mechanisms among the above possible origins remains a formidable challenge. In particular, identifying the main ET process responsible for red UCL among the possible ET processes is particularly difficult [17,18].

In recent years, efforts of changing the excitation wavelength for UCL materials have been devoted, owing to the high risk for human eyes [19] and the overheating effect for biological applications [20] of 980 nm excitation. Typically, using Nd^3+^ as sensitizer to replace Yb^3+^ can switch the excitation wavelength to 800 nm. Nd^3+^ sensitized UCL materials boost great research interests due to their strong energy harvest and deep penetration in biological tissues [21]. However, the Nd^3+^-sensitized materials usually require complex structures to achieve high UCL efficiency [22,23].

Alternatively, excitation at ~1.5 μm shows great potential for Er^3+^ singly doped UCL materials with simple structures, mainly due to the following reasons: First, 1.5 μm excitation shows less scattering loss than that of 980 nm excitation in biological tissues. Second, the Er^3+ 4^I_13/2_ state has a large absorption cross section at 1.5 μm [24], enabling the efficient energy harvest. Third, the lifetime of Er^3+ 4^I_13/2_ state exceeds 10 ms [25,26] and the unique 4f electron configuration of Er^3+^ enables the successive excited-state absorption (ESA) of 1.5 μm photons, validating further populations of Er^3+^ high energy states.

To date, Er^3+^ self-sensitized UCL in oxides [27], fluorides [28,29,30,31,32,33], and other compounds [34,35,36,37,38,39] have exhibited high efficiency upon ~1.5 μm excitation. However, similar to the situation in 980 nm excited Er^3+^ UCL materials, it is quite difficult to clarify the luminescent mechanisms, especially for the red emission. For instance, the origins of Er^3+^ self-sensitized red UCL upon 1.5 μm excitation were generally attributed to the following processes solely or synergistically: ESA from ^4^I_11/2_ [27,29,30,34,35,39], ET between ^2^H_11/2_ and ^4^I_11/2_ [31], ET between ^4^I_11/2_ and ^4^I_13/2_ [32,33], and nonradiative decay from ^4^S_3/2_ [36,37].

Clarifying the UCL mechanisms of Er^3+^ upon 0.98 and 1.5 μm excitation is crucial for fully exploiting the potentials of Er^3+^-doped UCL materials. However, current literature discussing the UCL mechanisms of Er^3+^ upon excitation with various wavelengths (typically 980 nm and 1.5 μm), generally either suffer from weak evidence [40,41], or require elaborated and time-consuming numerical simulations [9,42]. In this paper, different concentrations of Er^3+^-doped *β*-NaYF_4_ are synthesized. The UCL mechanisms of the samples via 980 and 1530 nm excitation are discussed on the basis of the spectroscopic data, and the discussion is further verified using simplified rate equation models.

## 2. Materials and Methods

### 2.1. Materials

Re(NO_3_)_3_∙6H_2_O (Re = Y and Er, 99.99%), NaF (98%), and ethylenediamine tetraacetic acid (EDTA, 99.5%) were purchased from Aladdin Co. Ltd., Shanghai, China. Ethanol (99.7%) was provided by Macklin Co. Ltd., Shanghai, China. All chemicals were used as received. 

### 2.2. Synthesis

The hexagonal NaYF_4_ (*β*-NaYF_4_) doped with *x* mol% Er^3+^, *x* = 2, 5, 10, 20, 30, and 40, were prepared by a modified hydrothermal method [43], and were thereafter denoted as *x*Er samples. In a typical route, Re(NO_3_)_3_∙6H_2_O (Er + Y = 4 mmol) with pre-determined ratios were first dissolved into 20 mL of deionized water after stirring for 30 min. Then, an aqueous solution of NaF (50 mmol, 10 mL) was added, and the mixture was kept stirring for 30 min. Afterwards, 4 mmol of EDTA, together with 30 mL of deionized water, were added and stirred for 1 h at room temperature. The resulting mixtures were transferred into Teflon-lined autoclaves and heated up to 200 °C for 20 h. After cooling down to room temperature, the reacting product was collected by centrifugation and washed with ethanol and deionized water several times, and dried at 80 °C for 20 h in air. For future spectral measurements, all the powder form samples were pressed into smooth plates, using an identical pressing setting.

### 2.3. Characterization

The crystallite structures of the as-prepared samples were identified by X-ray diffraction (XRD, XRD-6100, Shimadzu, Kyoto, Japan) measurements. The morphologies of the samples were recorded via a transmission electron microscope (TEM, Tecnai G2, FEI, Hillsboro, OH, USA). Room temperature luminescence measurements were performed by irradiating the samples via variable-power NIR diode lasers (LWIRL980-5W and LWIRL1530-1W, Laserwave, Beijing, China), with an excitation beam spot of around 1 mm^2^. The steady-state and time-resolved photoluminescence curves were measured by a customized ultraviolet to mid-infrared steady-state and phosphorescence lifetime spectrometer (QM8000, Horiba, Beijing, China) equipped with a tunable midband OPO laser as the pulse excitation source (410–2400 nm, Vibrant 355II, OPOTEK, Carlsbad, CA, USA). To validate the spectral comparisons, samples in powder form were ground and then pressed into round disks with two smooth surfaces. The usage of powders, pressing pressure, and diameter and thickness of the disks was identical.

## 3. Results

### 3.1. Structure Characterization

**Figure 2:**
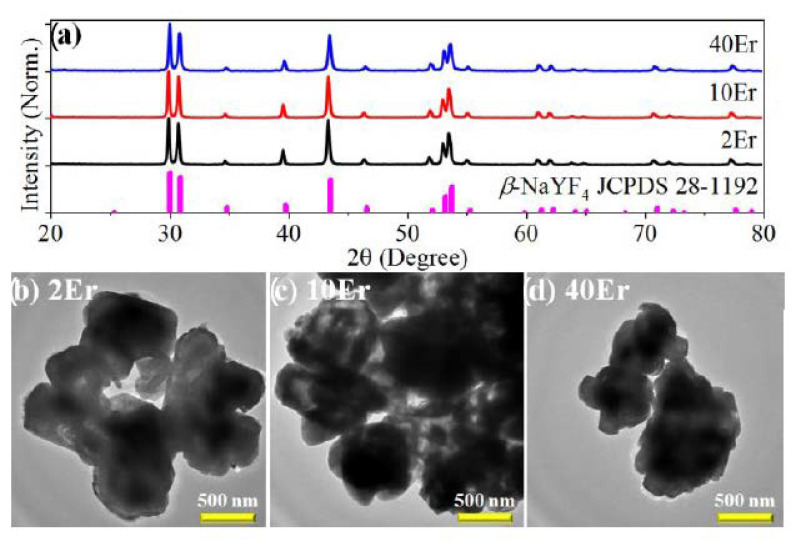
Structural characterizations of typical samples. (**a**) XRD patterns with standard diffraction data of β-NaYF4 as a reference; (**b**–**d**) TEM images of the samples. Scale bars are all 500 nm.

Figure 2a shows the XRD patterns of the as-prepared samples and the standard diffraction data of *β*-NaYF_4_ (JCPDS No. 28-1192). Three typical concentrations, representing low (2 mol%), moderate (10 mol%), and high (40 mol%) doping levels, were used for the XRD tests. All the diffraction peaks of the sample are consistent with the standard data and no obvious diffraction peaks of other impurities are observed, indicating the high purity of the hexagonal crystallite structure of samples. 

Figure 2b–d show the TEM images of 2Er, 10Er, and 40Er samples. It can be seen that all samples are irregular blocks with sizes of typically 10^2^ nm, and no substantial difference appears in these samples. Although it is well known that the particle size and shape of NaReF_4_ are sensitive to the type and concentration of the dopants, the morphologies of all samples are highly similar in the current case, which might be attributed to the similar ionic radii of Y^3+^ (0.90 Å) and Er^3+^ (0.89 Å). Due to the unchanged morphology, we can exclude the effects of the morphology when comparing the intrinsic UCL properties among different samples.

It is noteworthy that the as-prepared samples are not nanorods, which is the typical morphology of the NaReF_4_ nanomaterials prepared through a hydrothermal route. The formation of the irregular blocks rather than regular microrods might be due to the relatively higher synthesis temperature as well as relatively longer synthesis time, which lead the particles to dissolve and aggregate, similar to the morphology evolution of NaReF_4_ hydrothermally prepared elsewhere [44].

### 3.2. Luminescent Properties 

**Figure 3:**
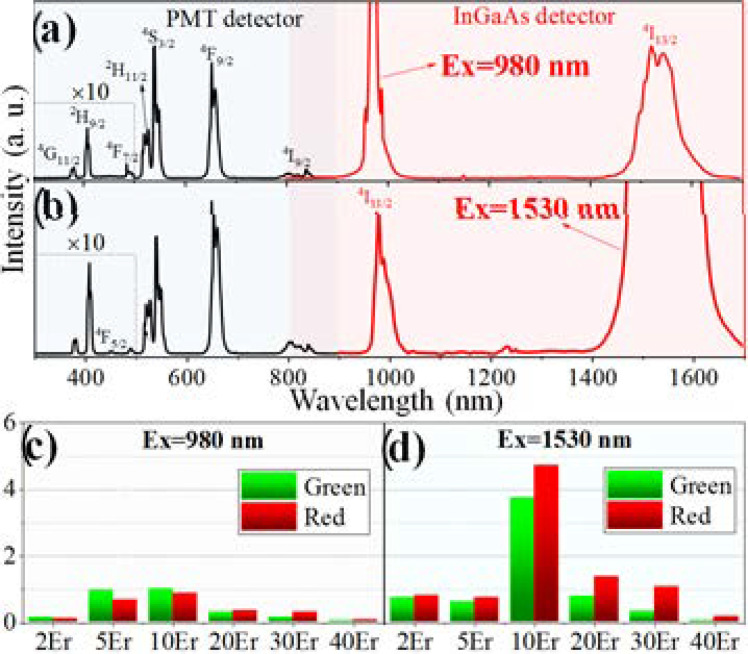
Photoluminescence spectra of 10Er sample upon (**a**) 980 and (**b**) 1530 nm excitation. The spectra in the wavelength range of 300~500 nm were enlarged by a factor of 10, for the sake of clarity; (**c**,**d**) are the histograms of the overall intensities of green and red UC emissions of different samples upon 980 and 1530 nm excitation, respectively, normalized by the green intensity of the 10Er sample.

The typical UCL spectra—using the 10Er sample as a representative as it is the most efficient—upon 980 and 1530 nm excitations were shown in Figure 3, in which an identical excitation power density of 100 W/cm^2^ was used for both excitation sources. Figure 3a shows the emission spectra upon 980 nm excitation, the 300~900 nm spectra were recorded by a PMT detector, while NIR spectrum ranging 800~1700 nm were recorded by an InGaAs detector. Eight characteristic emission bands of Er^3+^ can be observed. Emission peaks at 381, 408, 490, 520, 541, 654, 807, and 1532 nm can be attributed to the transitions of ^4^G_11/2_, ^2^H_9/2_, ^4^F_7/2_, ^2^H_11/2_, ^4^S_3/2_, ^4^F_9/2_, ^4^I_9/2_, and ^4^I_13/2_ state to the ground state ^4^I_15/2_, respectively. The transition of ^4^I_11/2_⟶^4^I_15/2_ overlaps with the excitation laser line, and thereby cannot be clearly seen. From another side, switching the excitation wavelength to 1530 nm induces ^4^I_11/2_⟶^4^I_15/2_ transition, centered at 980 nm. In addition, another emission band previously absent, centered at 450 nm corresponding to ^4^F_5/2_⟶^4^I_15/2_ transition, also appears upon 1530 nm excitation (Figure 3b).

Notably, the UCL intensity of the 10Er sample is stronger when using 1530 nm excitation compared to that of 980 nm excitation. Actually, 1530 nm excitation generally yields more intense UCL in samples doped with different Er^3+^ concentrations. Figure 3c,d show the integral intensities of green and red UCL of *x*Er samples upon different excitations. Except the 5Er sample, which is somehow weak, all others exhibit higher UCL intensity when using 1530 nm excitation. The general improvements in UCL intensity by using 1530 nm excitation can mainly stem from the stronger energy harvest of Er^3+^ at this wavelength [24], as well as the longer lifetime of ^4^I_13/2_ state [45,46]. The brightest UCL were obtained in the 10Er sample for both 980 and 1530 nm excitation, and the enhanced factors of green and red emission via 1530 nm excitation reach to around 4 and 5, respectively, compared to that of 980 nm excitation. The first increase and then decrease in the overall UCL intensity with the doping concentration might be related to the competition between energy harvest (positively correlates to the concentration) and concentration quenching effect (negatively correlates to the concentration). Another feature is that the red to green intensity ratios both increase with increasing Er^3+^ concentration for two excitations, suggesting concentration-dependent populations for the red state ^4^F_9/2_. The concentration-dependent population of the red state is stronger when using 1530 nm excitation, as evidenced by the larger red to green ratio obtained in the same sample upon different excitations. It is noteworthy that red light generally achieves deeper penetration than green light in biological tissues. Thus, the strong red UCL of the 10Er sample upon 1530 nm excitation may be of use in the in vivo applications.

**Figure 4:**
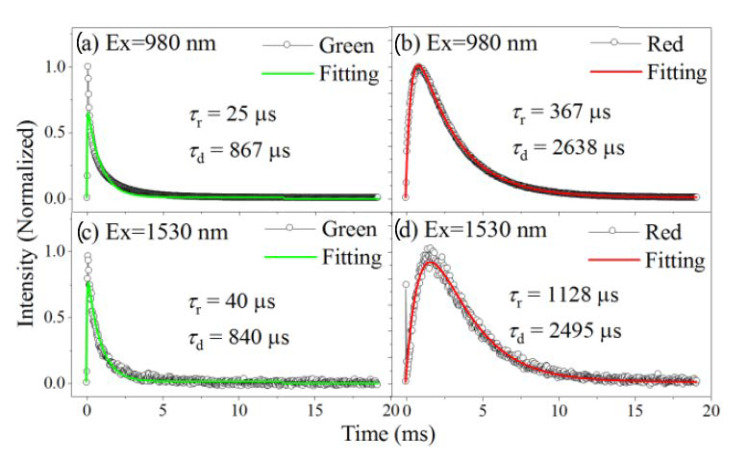
Time-resolved UCL of (**a**) green and (**b**) red emission upon 980 nm excitation and (**c**) green and (**d**) red emission upon 1530 nm excitation of the 10Er sample. The fitting curves, as well as the rise- and decay-times, are presented.

To investigate the population and decay processes of Er^3+^ UCL, we record the time-resolved UCL of the 10Er sample upon pulse excitations, which are further modeled using a reported method [47]. For the population processes after pulse 980 nm excitation, green UCL rapidly reaches its maximum (25 µs rise-time as shown in Figure 4a), while red UCL increases gradually (367 µs rise-time as shown in Figure 4b), leading to an obvious delayed onset time of the red decay. The rapid and relatively slow populations indicate that the ESA and ETU are responsible for the populations of green and red UCL, respectively. Once switching the pulse excitation wavelength to 1530 nm, the Er^3+^ green population is slightly prolonged, with a rise-time of ~40 μs (Figure 4c). This prolonged process indicates that the ETU start to play roles in the green population when using 1530 nm excitation. In addition, a large rise-time as high as ~1128 μs appears for the red UCL (Figure 4d), which clearly manifests the different origins of red UCL upon 980 and 1530 nm excitation. 

As for the decay processes, Er^3+^ green and red UCL both remain substantially unchanged when using different excitation wavelengths, due to the decay pathways being less dependent on the excitation wavelengths. Notably, the red emissions decay is evidently slower than the green emissions, for both 980 and 1530 nm excitation. This can be mainly attributed to the combination of radiative and nonradiative decay behaviors. From one hand, the radiative transition rates of Er^3+^ green (10^3^ s^−1^ for ^4^S_3/2_/^2^H_11/2_) and red (10^2^ s^−1^ for ^4^F_9/2_) emissions vary considerably [48], which partially contributes to the difference of green and red UCL decay-times. From another hand, nonradiative decay from upper state (^4^F_7/2_) to green states (^4^S_3/2_/^2^H_11/2_) is extremely fast, while the nonradiative decay that feeds the red state (^4^S_3/2_→^4^F_9/2_) is relatively slow, also leading to the prolonged decay-time of red UCL. It was noted that the nonradiative decay rates are similar for Er^3+^ green and red states as they have similar energy gaps of ~3000 cm^−1^ to their lower neighboring states, and thus are unlikely to be responsible for the varied decay-times. 

On the basis of the above discussions, we propose the following mechanisms responsible for Er^3+^ UCL: the population of green emission state upon 980 nm excitation can stem from the ESA, due to the green UCL immediately increasing to its maximum after pulse excitation. The ETU becomes the dominant populating process for Er^3+^ green UCL when using 1530 nm excitation, as evidenced by the prolonged population (Figure 4c). It has been reported that ESA tends to dominate the UCL process in low doping samples, while ETU is mainly responsible for the UCL processes in high doping samples [49], due to the stronger ET in high doping situations. In the current case, the stronger absorption of Er^3+^ at 1530 nm compared to that at 980 nm [24] results in stronger population in the intermediate state, and thus the stronger ET. 

From another side, the red population originates from the ET process for both 980 and 1530 nm excitation, which is consistent with the evidently prolonged population of red UCL (Figure 4b). For 980 nm excitation, we assume that the dominant ET process for the red UCL is between ^4^F_7/2_ and ^4^I_11/2_ states, while ET between ^4^I_11/2_ and ^4^I_13/2_ is mainly responsible for the 1530 nm excited red UCL. These assumptions can well explain why the population of red UCL can be further prolonged by 1530 nm excitation, since the lifetime of ^4^I_13/2_ state is much larger than that of the ^4^F_7/2_ state.

**Figure 5:**
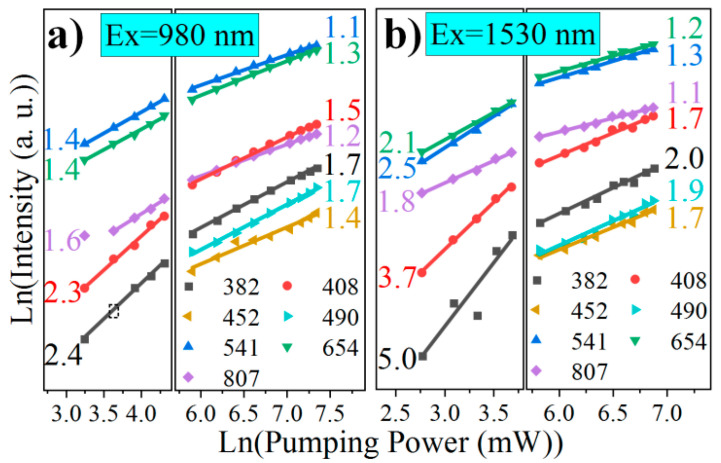
Measured emission intensities from Er^3+ 4^G_11/2_, ^2^H_9/2_, ^4^F_5/2_, ^4^F_7/2_, ^4^S_3/2_, ^4^F_9/2_, and ^4^I_9/2_ levels at 382, 408, 452, 490, 541, 654, and 807 nm, respectively, in the 10Er sample versus the pumping powers of (**a**) 980 and (**b**) 1530 nm excitation. The numbers denote the slopes of the power dependencies.

To further clarify the UCL mechanisms of Er^3+^ upon 1530 nm excitation, the variations of different peak intensities with the pumping power, i.e., the power dependences, are measured. The power dependences at 452 and 490 nm are absent in the low pumping region, due to the extremely weak light signals. As shown in Figure 5, all the ln-ln UCL power dependences can be well fitted linearly, but separated into two regions with increasing pumping power. The slopes of the linear fitting lines in the low pumping power region are obviously larger than that in the high pumping power region. The slopes derived from the power dependences under the weak pumping, capable of representing the photon numbers involved in an UCL process, are widely investigated [50,51,52]. In stark contrast, high pumping slopes are rarely paid attention, although they deliver important information as well. 

### 3.3. Modeling the Upconversion Luminescence Processes

**Figure 6:**
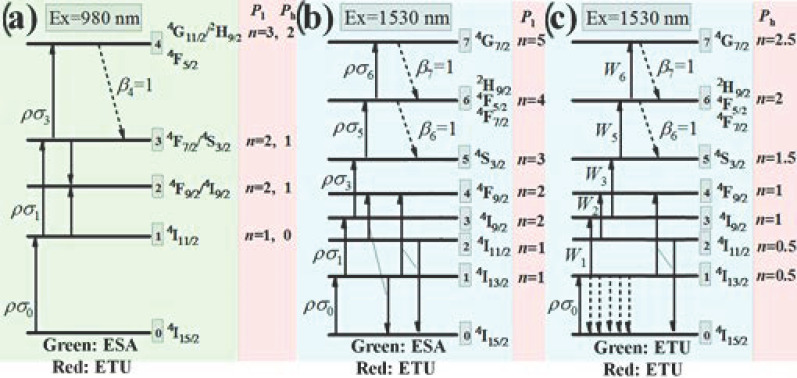
Simplified energy levels of Er^3+^ with the dominant upconversion pathways upon (**a**) 980 nm excitation, (**b**) weak 1530 nm excitation, and (**c**) strong 1530 nm excitation.

We further set up simplified rate equations to calculate the theoretical slopes of the power dependences of Er^3+^ UCL, using a five- (Figure 6a) and eight-energy-level (Figure 6b,c) model for 980 and 1530 nm excitation, respectively. The details of the establishment of the rate equations, as well as the extraction of the slopes, refer to the reports elsewhere [49].

#### 3.3.1. Excitation at 980 nm

As mentioned above, we assume the ESA and ETU processes dominate Er^3+^ green and red UCL mechanisms upon 980 nm excitation, respectively (Figure 6a). The corresponding rate equations can be given as follows:

(1) $$\begin{array}{l} \begin{array}{cc} 1: & \rho \sigma_{0}N_{0}-\rho \sigma_{1}N_{1}-WN_{1}N_{3}-A_{1}N_{1}+\beta_{2}A_{2}N_{2}=0 \end{array}\\ \begin{array}{cc} 2: & 2WN_{1}N_{3}+\beta_{3}A_{3}N_{3}-A_{2}N_{2}=0 \end{array}\\ \begin{array}{cc} 3: & \rho \sigma_{1}N_{1}-\rho \sigma_{3}N_{3}-WN_{1}N_{3}+\beta_{4}A_{4}N_{4}-A_{3}N_{3}=0 \end{array}\\ \begin{array}{cc} 4: & \rho \sigma_{3}N_{3}-A_{4}N_{4}=0 \end{array} \end{array}$$

where *N_i_*, *σ_i_*, *ρ*, *W*, *A_i_*, and *β_i_* stands for the population density of level *i* (*i* = 0, 1, 2, 3, and 4); absorption cross section for level *i*; pumping rate (proportional to incident laser power); ET rate between energy levels 1 and 3; transition rate of level *i*, including the radiative transition to the ground state and the multiphonon-assisted decay to its lower level; and fraction of the multiphonon-assisted decay rate, respectively. 

For the 980 nm weak pumping situation, the downward decay *A_i_N_i_* dominates the depopulation of every state, then we obtain:

(2) $$\begin{array}{cc} \begin{array}{l} \begin{array}{cc} 1: & \rho \sigma_{0}N_{0}-A_{1}N_{1}=0 \end{array}\\ \begin{array}{cc} 2: & \beta_{3}A_{3}N_{3}-A_{2}N_{2}=0 \end{array}\\ \begin{array}{cc} 3: & \rho \sigma_{1}N_{1}-A_{3}N_{3}=0 \end{array}\\ \begin{array}{cc} 4: & \rho \sigma_{3}N_{3}-A_{4}N_{4}=0 \end{array} \end{array} & \begin{array}{cc} \Rightarrow & \begin{array}{l} N_{1}\propto P^{1}\\ N_{2}\propto P^{2}\\ N_{3}\propto P^{2}\\ N_{4}\propto P^{3} \end{array} \end{array} \end{array}$$

For 980 nm strong pumping, as the ESA and ET processes increase more evidently with the incident laser power than the multiphonon-assisted decay process, we assume that the upward ESA (*ρσ*_1_*N*_1_) dominates the depopulation of energy level 1, and the ET process dominates the depopulation of energy level 3 (*WN*_1_*N*_3_ >> *β*_3_*A*_3_*N*_3_). In addition, the fraction *β*_4_ is set to be 1, due to the closely distributed states of Er^3+^ in the higher energy region. We therefore obtain:

(3) $$\begin{array}{ccc} \begin{array}{l} \begin{array}{cc} 1: & \rho \sigma_{0}N_{0}-\rho \sigma_{1}N_{1}=0 \end{array}\\ \begin{array}{cc} 2: & 2WN_{1}N_{3}-A_{2}N_{2}=0 \end{array}\\ \begin{array}{cc} 3: & \rho \sigma_{1}N_{1}-WN_{1}N_{3}-A_{3}N_{3}=0 \end{array}\\ \begin{array}{cc} 4: & \rho \sigma_{3}N_{3}-A_{4}N_{4}=0 \end{array} \end{array} & \Rightarrow & \begin{array}{l} N_{1}\propto P^{0}\\ N_{2}\propto P^{1}\\ N_{3}\propto P^{1}\\ N_{4}\propto P^{2} \end{array} \end{array}$$

From above, it can be concluded that the slope values *n* stand for the photon numbers involved in the corresponding UCL processes in the weak pumping situation, and strong pumping results in slope values decreasing to *n* − 1. 

The slope values derived (*n*_Ideal_) and the slope values measured (*n*_Real_) are summarized in Table 1. All the *n*_Ideal_ are close to *n*_Real_, with only slight deviations. The slight deviations of *n*_Ideal_ to the integers may stem from the competition between upward and downward transition which depopulates the intermediate state, as the integers were derived on the basis of assumption under the extreme situations. Only one large deviation upon high power 980 nm excitation appears at 490 nm (*n*_Real_ = 1.7 and *n*_Ideal_ = 1). This exception can stem from the thermal coupling effects, which leads to the higher level exhibiting a larger slope value [43]. The evolution of slope values of ^4^F_7/2_, ^2^H_11/2_, and ^4^S_3/2_ state (1.68→1.56 (data not shown)→1.10) also supports this conclusion. These three states, with energy gaps below 2000 cm^−1^ validating the effective thermal couples among them, show increased slopes with increasing their energy.

#### 3.3.2. Excitation at 1530 nm—Weak Pumping

To investigate the Er^3+^ UCL mechanisms upon weak 1530 nm excitation, a more complicated model with eight energy levels is adopted as shown in Figure 6b. Similar to the situation of 980 nm excitation, we assume the ESA and ETU processes dominate the green and red UCL mechanisms, respectively. However, the ETU responsible for the red UCL of Er^3+^ upon 1530 nm excitation switches to ET between energy levels 1 and 2. Two possible ETU processes between levels 1 and 2, as labeled in Figure 5b, can be expressed by an identical term of *W*_2_*N*_1_*N*_2_, where *W*_2_ is the ET rate. The previously established ET process between energy levels 2 and 5 is absent in this model, mainly due to the fact that the energy level 1 should be populated more strongly as compared to that of level 5, when Er^3+^ is excited at 1530 nm. The corresponding rate equations can be given as follows:

(4) $$\begin{array}{l} \begin{array}{cc} 1: & \rho \sigma_{0}N_{0}-\rho \sigma_{1}N_{1}-W_{2}N_{1}N_{2}+\beta_{2}A_{2}N_{2}-A_{1}N_{1}=0 \end{array}\\ \begin{array}{cc} 2: & \beta_{3}A_{3}N_{3}-W_{2}N_{1}N_{2}-A_{2}N_{2}=0 \end{array}\\ \begin{array}{cc} 3: & \rho \sigma_{1}N_{1}-\rho \sigma_{3}N_{3}+\beta_{4}A_{4}N_{4}-A_{3}N_{3}=0 \end{array}\\ \begin{array}{cc} 4: & W_{2}N_{1}N_{2}+\beta_{5}A_{5}N_{5}-A_{4}N_{4}=0 \end{array}\\ \begin{array}{cc} 5: & \rho \sigma_{3}N_{3}-\rho \sigma_{5}N_{5}+\beta_{6}A_{6}N_{6}-A_{5}N_{5}=0 \end{array}\\ \begin{array}{cc} 6: & \rho \sigma_{5}N_{5}-\rho \sigma_{6}N_{6}+\beta_{7}A_{7}N_{7}-A_{6}N_{6}=0 \end{array}\\ \begin{array}{cc} 7: & \rho \sigma_{6}N_{6}-A_{7}N_{7}=0 \end{array} \end{array}$$

**Figure 7:**
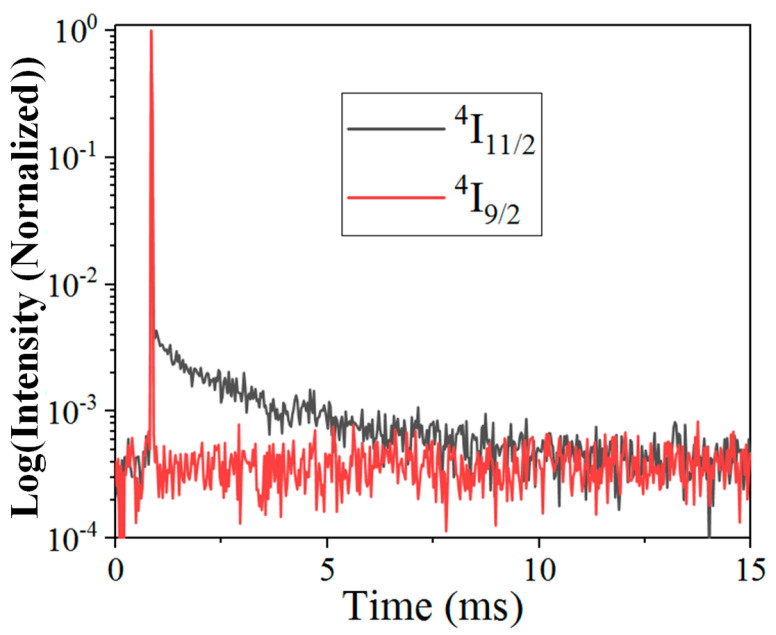
Time−resolved Er^3+^ emissions at 800 nm (^4^I_9/2_) and 980 nm (^4^I_11/2_) via pulse 795 nm and pulse 975 nm excitation, respectively.

For the 1530 nm weak pumping situation, the downward decay *A_i_N_i_* dominates the depopulation mechanisms of every energy level, except level 2. The dominant depopulation mechanism of level 2 is assumed to be ETU between levels 1 and 2, *W*_2_*N*_1_*N*_2_. On one hand, strong decay from higher level 3, after ESA, can effectively populate level 2; on the other hand, the larger energy gap of level 2 to its lower level suppresses the downward decay. As shown in Figure 7, the rapid and slow decay of Er^3+ 4^I_9/2_ (level 3) and ^4^I_11/2_ (level 2), respectively, evidence the above-mentioned strong decay from level 3 and weak decay from level 2. Therefore, strong populations of levels 1 and 2 enable their efficient ET even in the weak pumping situation. Further, the corresponding rate equations can be derived as follows:

(5) $$\begin{array}{ccc} \begin{array}{l} \begin{array}{cc} 1: & \rho \sigma_{0}N_{0}-A_{1}N_{1}=0 \end{array}\\ \begin{array}{cc} 2: & \beta_{3}A_{3}N_{3}-W_{2}N_{1}N_{2}=0 \end{array}\\ \begin{array}{cc} 3: & \rho \sigma_{1}N_{1}-A_{3}N_{3}=0 \end{array}\\ \begin{array}{cc} 4: & W_{2}N_{1}N_{2}-A_{4}N_{4}=0 \end{array}\\ \begin{array}{cc} 5: & \rho \sigma_{3}N_{3}-A_{5}N_{5}=0 \end{array}\\ \begin{array}{cc} 6: & \rho \sigma_{5}N_{5}-A_{6}N_{6}=0 \end{array}\\ \begin{array}{cc} 7: & \rho \sigma_{6}N_{6}-A_{7}N_{7}=0 \end{array} \end{array} & \Rightarrow & \begin{array}{l} N_{1}\propto P^{1}\\ N_{2}\propto P^{1}\\ N_{3}\propto P^{2}\\ N_{4}\propto P^{2}\\ N_{5}\propto P^{3}\\ N_{6}\propto P^{4}\\ N_{7}\propto P^{5} \end{array} \end{array}$$

In the weak pumping region, as shown in Table 1, the *n*_Ideal_ match well with the *n*_Real_. The slight deviations can also stem from the competition between upward and downward transitions. However, using this model cannot derive convincing results for the strong pumping situation (data not shown), indicating the different mechanisms of Er^3+^ UCL when the sample is strongly pumped at 1530 nm.

#### 3.3.3. Excitation at 1530 nm—Strong Pumping

For Er^3+^ upon strong 1530 nm excitation, a similar model as used in the weak excitation situation is set up. As shown in Figure 6c, the only difference between this model and the previous one is that the ETU rather than the ESA dominates the UCL mechanisms, as assumed before. The corresponding rate equations can be given as follows:

(6) $$\begin{array}{l} \begin{array}{cc} 1: & \rho \sigma_{0}N_{0}-2W_{1}N_{1}N_{1}-\sum_{i=2,3,5,6}W_{i}N_{1}N_{i}+\beta_{2}A_{2}N_{2}-A_{1}N_{1}=0 \end{array}\\ \begin{array}{cc} 2: & \beta_{3}A_{3}N_{3}-W_{2}N_{1}N_{2}-A_{2}N_{2}=0 \end{array}\\ \begin{array}{cc} 3: & W_{1}N_{1}N_{1}-W_{3}N_{1}N_{3}+\beta_{4}A_{4}N_{4}-A_{3}N_{3}=0 \end{array}\\ \begin{array}{cc} 4: & W_{2}N_{1}N_{2}+\beta_{5}A_{5}N_{5}-A_{4}N_{4}=0 \end{array}\\ \begin{array}{cc} 5: & W_{3}N_{1}N_{3}-W_{5}N_{1}N_{5}+\beta_{6}A_{6}N_{6}-A_{5}N_{5}=0 \end{array}\\ \begin{array}{cc} 6: & W_{5}N_{1}N_{5}-W_{6}N_{1}N_{6}+\beta_{7}A_{7}N_{7}-A_{6}N_{6}=0 \end{array}\\ \begin{array}{cc} 7: & W_{6}N_{1}N_{6}-A_{7}N_{7}=0 \end{array} \end{array}$$

For strong pumping, one can further obtain:

(7) $$\begin{array}{ccc} \begin{array}{l} \begin{array}{cc} 1: & \rho \sigma_{0}N_{0}-2W_{1}N_{1}N_{1}=0 \end{array}\\ \begin{array}{cc} 2: & \beta_{3}A_{3}N_{3}-W_{2}N_{1}N_{2}=0 \end{array}\\ \begin{array}{cc} 3: & W_{1}N_{1}N_{1}-A_{3}N_{3}=0 \end{array}\\ \begin{array}{cc} 4: & W_{2}N_{1}N_{2}-A_{4}N_{4}=0 \end{array}\\ \begin{array}{cc} 5: & W_{3}N_{1}N_{3}-A_{5}N_{5}=0 \end{array}\\ \begin{array}{cc} 6: & W_{5}N_{1}N_{5}-A_{6}N_{6}=0 \end{array}\\ \begin{array}{cc} 7: & W_{6}N_{1}N_{6}-A_{7}N_{7}=0 \end{array} \end{array} & \Rightarrow & \begin{array}{l} N_{1}\propto P^{1/2}\\ N_{2}\propto P^{1/2}\\ N_{3}\propto P^{1}\\ N_{4}\propto P^{1}\\ N_{5}\propto P^{3/2}\\ N_{6}\propto P^{2}\\ N_{7}\propto P^{5/2} \end{array} \end{array}$$

As shown in Table 1, all the derived slope values *n*_Ideal_ are in good agreement with their corresponding practical values *n*_Real_, verifying the reliability of the modeling.

## 4. Conclusions

Er^3+^-doped NaYF_4_ were synthesized by a hydrothermal method. It is found that the 10Er sample yields the most efficient UCL upon both 980 and 1530 nm excitation, and 1530 nm excitation induces a more than four-fold stronger UCL as compared to that upon 980 nm excitation, mainly due to the much stronger energy harvest of Er^3+^ at ~1.5 μm, as well as the longer lifetime of Er^3+ 4^I_13/2_ state. For 980 nm excitation (weak or strong), the main mechanisms responsible for green and red UCL are ESA and ETU in higher energy regions (^4^F_7/2_/^2^H_11/2_/^4^S_3/2_ + ^4^I_11/2_→^4^F_9/2_ + ^4^F_9/2_), respectively. As for 1530 nm excitation, the green UCL are mainly induced by ESA and ETU upon weak and strong pumping, respectively, while ETU is the dominant origin of the red UCL upon both weak and strong pumping. Notably, it is proposed that the ETU between lower energy levels, ^4^I_11/2_ + ^4^I_13/2_→^4^F_9/2_ + ^4^I_15/2_, is dominant when using 1530 nm excitation.

## Figures and Tables

**Table 1:** Summarized power dependences data of the 10Er sample, where *i* stands for the photon number required for populating the UCL energy level; *λ* is the emission peak; and *P*_l_ and *P*_h_ represent low pumping and high pumping, respectively.

*i*	Ex = 980 nm				Ex = 1530 nm			
	Transition	*λ* (nm)	*n*_Real_ *P*_l_→*P*_h_	*n*_Ideal_ *P*_l_→*P*_h_	Transition	*λ* (nm)	*n*_Real_ *P*_l_→*P*_h_	*n*_Ideal_ *P*_l_→*P*_h_
2	^4^I_9/2_→^4^I_15/2_	807	1.6→1.2	2→1	^4^I_9/2_→^4^I_15/2_	807	1.8→1.1	2→1
	^4^F_9/2_→^4^I_15/2_	654	1.4→1.3					
	^4^S_3/2_→^4^I_15/2_	541	1.4→1.1					
	^4^F_7/2_→^4^I_15/2_	490	NA→**1.7**					
3	^4^F_5/2_→^4^I_15/2_	452	NA→1.4	3→2	^4^F_9/2_→^4^I_15/2_ ^4^S_3/2_→^4^I_15/2_	654 541	**2.1**→1.2 2.5→1.3	2→1.5 3→1.5
	^2^H_9/2_→^4^I_15/2_	408	2.3→1.5					
	^4^G_11/2_→^4^I_15/2_	382	2.4→1.7					
4					^4^F_7/2_→^4^I_15/2_	490	NA→1.9	4→2
					^4^F_5/2_→^4^I_15/2_	452	NA→1.7	
					^2^H_9/2_→^4^I_15/2_	408	3.7→1.7	
5					^4^G_11/2_→^4^I_15/2_	382	5.0→2.0	5→2.5

## Data Availability

The data presented in this study are available on request from the corresponding author. The data are not publicly available due to privacy.
